# The protocol of a prospective, multicenter, randomized, controlled phase III study evaluating different cycles of oxaliplatin combined with S-1 (SOX) as neoadjuvant chemotherapy for patients with locally advanced gastric cancer: RESONANCE-II trial

**DOI:** 10.1186/s12885-020-07764-7

**Published:** 2021-01-05

**Authors:** Xinxin Wang, Shuo Li, Yihong Sun, Kai Li, Xian Shen, Yingwei Xue, Pin Liang, Guoli Li, Luchuan Chen, Qun Zhao, Guoxin Li, Weihua Fu, Han Liang, Hairong Xin, Jian Suo, Xuedong Fang, Zhichao Zheng, Zekuan Xu, Huanqiu Chen, Yanbing Zhou, Yulong He, Hua Huang, Linghua Zhu, Kun Yang, Jiafu Ji, Yingjiang Ye, Zhongtao Zhang, Fei Li, Xin Wang, Yantao Tian, Sungsoo Park, Lin Chen

**Affiliations:** 1 Department of General Surgery & Institute of General Surgery, Chinese PLA General Hospital, No.28 Fuxing Road, Haidian District, Beijing, 100853 China; 2 Department of General Surgery, Zhongshan Hospital, Fudan University, No.180 Fenglin Road, Xuhui District, Shanghai, 200032 China; 3 Department of Surgical Oncology, The First Hospital of China Medical University, No.155 Nanjing Street North, Heping District, Shenyang, 110001 China; 4 Division of Gastrointestinal Surgery, The Second Affiliated Hospital of Wenzhou Medical University, No.109 West Xueyuan Road, Wenzhou, 325027 China; 5 Department of Gastroenterological Surgery, Harbin Medical University Cancer Hospital, No.150 Haping Road, Nangang District, Harbin, 150081 China; 6 Department of Gastrointestinal Surgery, The First Affiliated Hospital of Dalian Medical University, No.222 Zhongshan Road, Xigang District, Dalian, 116011 China; 7 Institute of General Surgery, General Hospital of Eastern Theater Command of Chinese PLA, No.305 East Zhongshan Road, Xuanwu District, Nanjing, 210002 China; 8 Department of Gastrointestinal Surgery, Fujian Cancer Hospital, Fujian Medical University Cancer Hospital, No.420 Fuma Road, Jinan District, Fuzhou, 350014 China; 9 Department of General Surgery, The Fourth Hospital of Hebei Medical University, No.12 Jiankang Road, Shijiazhuang, 050011 China; 10 Department of General Surgery, Nanfang Hospital, Southern Medical University, No.1838 Guangzhoudadaobei Road, Guangzhou, 510515 China; 11 Department of General Surgery, Tianjin Medical University General Hospital, No.154 Anshan Road, Heping District, Tianjin, 300052 China; 12 Department of Gastric Cancer Surgery, Tianjin Medical University Cancer Hospital, West Huan-Hu Road, Ti Yuan Bei, Hexi District, Tianjin, 300060 China; 13 Department of General Surgery, Shanxi Provincial Cancer Hospital, No.3 Zhigongxincun, Xinghualing District, Taiyuan, 030013 China; 14 Department of General Surgery, The First Bethune Hospital of Jilin University, No.71 Xinmindajie Street, Changchun, 130021 China; 15 Department of General Surgery, China-Japan Union Hospital of Jilin University, No.126 Xi’antai Avenue, Changchun, 130033 China; 16 Department of Gastric Surgery, Liaoning Cancer Hospital and Institute, No.44 Xiaoheyan Road, Dadong District, Shenyang, 110042 China; 17 Department of General Surgery, The First Affiliated Hospital of Nanjing Medical University, No.300 Guangzhou Road, Gulou District, Nanjing, 210029 China; 18 Department of General Surgery, Jiangsu Cancer Hospital (Jiangsu Institute of Cancer Research, Nanjing Medical University Affiliated Cancer Hospital), No.42 Baiziting, Nanjing, 210009 China; 19 Department of General Surgery, The Affiliated Hospital of Qingdao University, No.16 Jiangsu Road, Shinan District, Qingdao, 266003 China; 20 Department of Gastrointestinal Surgery, The First Affiliated Hospital, Sun Yat-sen University, No.58 Zhongshaner Road, Guangzhou, 510080 China; 21 Department of Gastric Surgery, Fudan University Shanghai Cancer Center, No.270 Dongan Road, Xuhui District, Shanghai, 200032 China; 22 Department of General Surgery, Sir Run Run Shaw Hospital, School of Medicine, Zhejiang University, No.3 East Qingchun Road, Jianggan District, Hangzhou, 310016 China; 23 Department of Gastrointestinal Surgery, West China Hospital, Sichuan University, No.37 Guoxue Alley, Wuhou District, Chengdu, 610041 China; 24 Department of Gastrointestinal Surgery, Peking University Cancer Hospital, No.52 Fucheng Road, Haidian District, Beijing, 100142 China; 25 Department of Gastroenterological Surgery, Peking University People’s Hospital, No.11 Xizhimen South Street, Xicheng District, Beijing, 100044 China; 26 Department of General Surgery, Beijing Friendship Hospital, Capital Medical University, No.95 Yongan Road, Xicheng District, Beijing, 100050 China; 27 Department of General Surgery, Xuanwu Hospital, Capital Medical University, No.45 Changchun Street, Xicheng District, Beijing, 100053 China; 28 Department of General Surgery, Peking University First Hospital, No.8 Xishiku Street, Xicheng District, Beijing, 100034 China; 29 Department of Pancreatic and Gastric Surgery, Cancer Hospital, Chinese Academy of Medical Sciences, No.17 Panjiayuannanli, Chaoyang District, Beijing, 100021 China; 30 Division of Upper GI Surgery, Department of Surgery, Korea University Anam Hospital, Korea University College of Medicine, 73 Goryeodae-ro Seongbuk-gu, Seoul, 02841 South Korea

**Keywords:** Locally advanced gastric cancer, Duration of neoadjuvant chemotherapy, S-1, Oxaliplatin

## Abstract

**Background:**

Curing locally advanced gastric cancer through surgery alone is difficult. Adjuvant and neoadjuvant chemotherapy bring potential benefits to more patients with gastric cancer based on several clinical trials. According to phase II studies and guidelines, SOX regimen as neoadjuvant chemotherapy is efficient. However, the optimal duration of neoadjuvant chemotherapy has not been established. In this study, we will evaluate the efficacy and safety of different cycles of SOX as neoadjuvant chemotherapy for patients with locally advanced gastric cancer.

**Methods:**

RESONANCE-II trial is a prospective, multicenter, randomized, controlled phase III study which will enroll 524 patients in total. Eligible patients will be registered, pre-enrolled and receive three cycles of SOX, after which tumor response evaluations will be carried out. Those who show stable disease or progressive disease will be excluded. Patients showing complete response or partial response will be enrolled and assigned into either group A for another three cycles of SOX (six cycles in total) followed by D2 surgery; or group B for D2 surgery (three cycles in total). The primary endpoint is the rate of pathological complete response and the secondary endpoints are R0 resection rate, three-year disease-free survival, five-year overall survival, and safety.

**Discussion:**

This study is the first phase III randomized trial to compare the cycles of neoadjuvant chemotherapy using SOX for resectable locally advanced cancer. Based on a total of six to eight cycles of perioperative chemotherapy usually applied in locally advanced gastric cancer, patients in group A can be considered to have completed all perioperative chemotherapy, the results of which may suggest the feasibility of using chemotherapy only before surgery in gastric cancer.

**Trial registration:**

Registered prospectively in the World Health Organization International Clinical Trials Registry Platform (WHO ICTRP) with registration number ChiCTR1900023293 on May 21st, 2019.

## Background

Gastric cancer remains the third leading cause of malignant tumor death both in China and worldwide [1, 2]. The overall five-year survival rate is about 20% [3]. At present radical gastrectomy is regarded as the only approach to curing gastric cancer. However, about 30% of patients with gastric cancer have recurrence after receiving radical gastrectomy [4, 5]. Thus it is difficult to achieve a cure for gastric cancer through surgery alone.

Several randomized controlled trials have evaluated the efficacy of adjuvant chemotherapy, that is, postoperative chemotherapy, for gastric cancer recently. The ACTS-GC phase III trial enrolled 1059 patients with stage II or III gastric cancer at 109 centers throughout Japan. They were assigned randomly to either the S-1 group for receiving S-1 as adjuvant chemotherapy for 1 year postoperatively or the surgery-only group for receiving surgery alone. The five-year overall survival rates in the S-1 group and surgery-only group were 71.7 and 61.1% respectively, and the five-year relapse-free survival rates were 65.4 and 53.1% respectively. S-1 reduced the risk of death by 33.1% and of relapse by 34.7% [6]. The CLASSIC trial enrolled 1035 patients at 35 cancer centers in South Korea, China Mainland and Taiwan. Patients with stage II-IIIB gastric cancer who underwent D2 radical gastrectomy were assigned randomly to adjuvant chemotherapy with eight cycles of capecitabine and oxaliplatin (XELOX) or observation alone. The results showed that 27% of patients in the adjuvant capecitabine and oxaliplatin group and 39% in the observation alone group had recurrence or died (*P*< 0.0001) and the estimated five-year disease-free survival rates were 68 and 53% respectively [7]. These two trials from Asia confirmed that adjuvant chemotherapy using S-1 or XELOX could improve survival in patients with gastric cancer who had received D2 gastrectomy.

Unlike in Asia, in western countries, neoadjuvant chemotherapy, that is, perioperative chemotherapy, received widespread interest. The MAGIC trial was the first study to confirm the efficacy of neoadjuvant chemotherapy in gastric cancer treatment. A total of 503 patients with resectable adenocarcinoma of the stomach, esophagogastric junction or lower esophagus were enrolled and then assigned to either the perioperative-chemotherapy group or surgery group. Three cycles of preoperative and three cycles of postoperative chemotherapy using epirubicin, cisplatin and fluorouracil (ECF) were administrated to patients only in the perioperative-chemotherapy group, which resulted in a higher five-year survival rate than that of the surgery group (36% vs 23%, *P*=0.009) [8]. Afterwards, the FNCLCC/FFCD 9703 phase III trial was conducted in France to investigate the benefit of perioperative fluorouracil plus cisplatin (CF) in resectable gastroesophageal adenocarcinoma. Two hundred twenty-four patients were randomly assigned to either the perioperative chemotherapy and surgery group (CS group) medicated with two or three cycles of CF before surgery and three or four cycles after surgery, or the surgery alone group (S group) for receiving surgery only. The results showed that the CS group had a better five-year overall survival (38% vs 24%, *P*=0.02) and five-year disease-free survival (34% vs 19%, *P*=0.003) and that the perioperative chemotherapy significantly improved the curative resection rate (84% vs 73%, *P*=0.04) [9]. However, the completion rate of perioperative chemotherapy in these two trials was relatively low. Later, the FLOT4 trial altered the situation. It aimed to compare the safety and efficacy of FLOT (fluorouracil plus leucovorin, oxaliplatin and docetaxel) with that of ECF/ECX (epirubicin, cisplatin and fluorouracil or capecitabine). Seven hundred sixteen patients were enrolled and randomly assigned to treatment in 38 German hospitals. Results showed that the median overall survival and chemotherapy completion rate were increased in the FLOT group (50 months vs 35 months, *P*=0.012; 46% vs 37%), which indicated that FLOT could be preferred compared to ECF/ECX [10].

Although all trials mentioned above had positive findings, the EORTC 40954 study, which was conducted in Europe, showed no survival benefit for neoadjuvant chemotherapy. This trial was for the purpose of proving the superiority of preoperative chemotherapy using cisplatin, d-L-folinic acid and fluorouracil. Compared with the surgery-only group, the neoadjuvant group had higher R0 resection rate (81.9% vs 66.7%, *P*=0.036) and less lymph node metastases (61.4% vs 76.5%, *P*=0.018). Survival benefit was not demonstrated after median follow-up of 4.4 years and 67 deaths (HR 0.84, 95% Cl 0.52 to 1.35, *P*=0.466). Notably, because of the poor accrual, the trial was terminated and only 144 patients were randomly assigned in the neoadjuvant arm or surgery-alone arm (72:72) instead of an estimated total of 360 patients. The low statistical power, the high rate of proximal gastric cancer and the better outcome than expected due to the high quality of surgery were possible reasons for no survival benefit [11].

Recently, S-1 plus oxaliplatin (SOX) as neoadjuvant chemotherapy showed relatively high efficacy and safety in several studies [12–14]. A phase II trial conducted by our medical center demonstrated that neoadjuvant chemotherapy with SOX regimen yielded an overall response rate of 68.8% and a disease control rate of 93.8%. The D2 lymph nodes dissection rate and R0 resection rate in neoadjuvant chemotherapy group were both higher than those in surgery-only group [15]. Furthermore, fluoropyrimidine plus oxaliplatin was recommended in the National Comprehensive Cancer Network (NCCN) Guidelines Version 1.2020 Gastric Cancer and the Chinese Society of Clinical Oncology (CSCO) guidelines for the diagnosis and treatment of gastric cancer (2019) [16, 17]. Therefore, the SOX is considered to have good application prospects.

It should be noted that the number of cycles of preoperative chemotherapy applied in these studies is not standardized and two to four cycles seemed to be acceptable based on previous studies [8–10]. So, the proper duration for neoadjuvant therapy needs to be settled. A randomized phase II trial conducted in Japan enrolled 83 patients with stage III and IV resectable gastric cancer. They were randomly assigned into two courses of SC (S-1 plus cisplatin), four courses of SC, two courses of PC (paclitaxel plus cisplatin) and four courses of PC. The study found no significant differences between two regimens (*P*=0.956) or between the two- and four- course treatments (*P*=0.723). However, the limitations of the study are the relatively small sample size and the inclusion of some stage IV patients, which could lead to the negative result [18].

Based on these findings, the randomized phase III RESONANCE-II trial was designed to evaluate the efficacy and safety of different cycles of SOX as neoadjuvant chemotherapy for patients with locally advanced gastric cancer, so as to provide a theoretical basis for setting an optimal duration of neoadjuvant chemotherapy.

## Methods

RESONANCE-II trial is a prospective, multicenter, randomized, controlled phase III study which will be conducted in 29 medical centers in China and one medical center in Korea. Chinese PLA General Hospital is the lead center. Lin Chen is the trial principal investigator (PI). All screened patients should state that they are healthy and have no discomfort, then they will sign the informed consent. After signing the informed consent, they will receive consultation from investigator and examinations. Eligible patients will be registered, pre-enrolled. Main possible reasons for screening failure include: (1) The results of physical examination or laboratory tests do not meet the inclusion criteria or meet the exclusion criteria; (2) Patients withdraw informed consent or refuse to participate in the research.

**Fig. 1:**
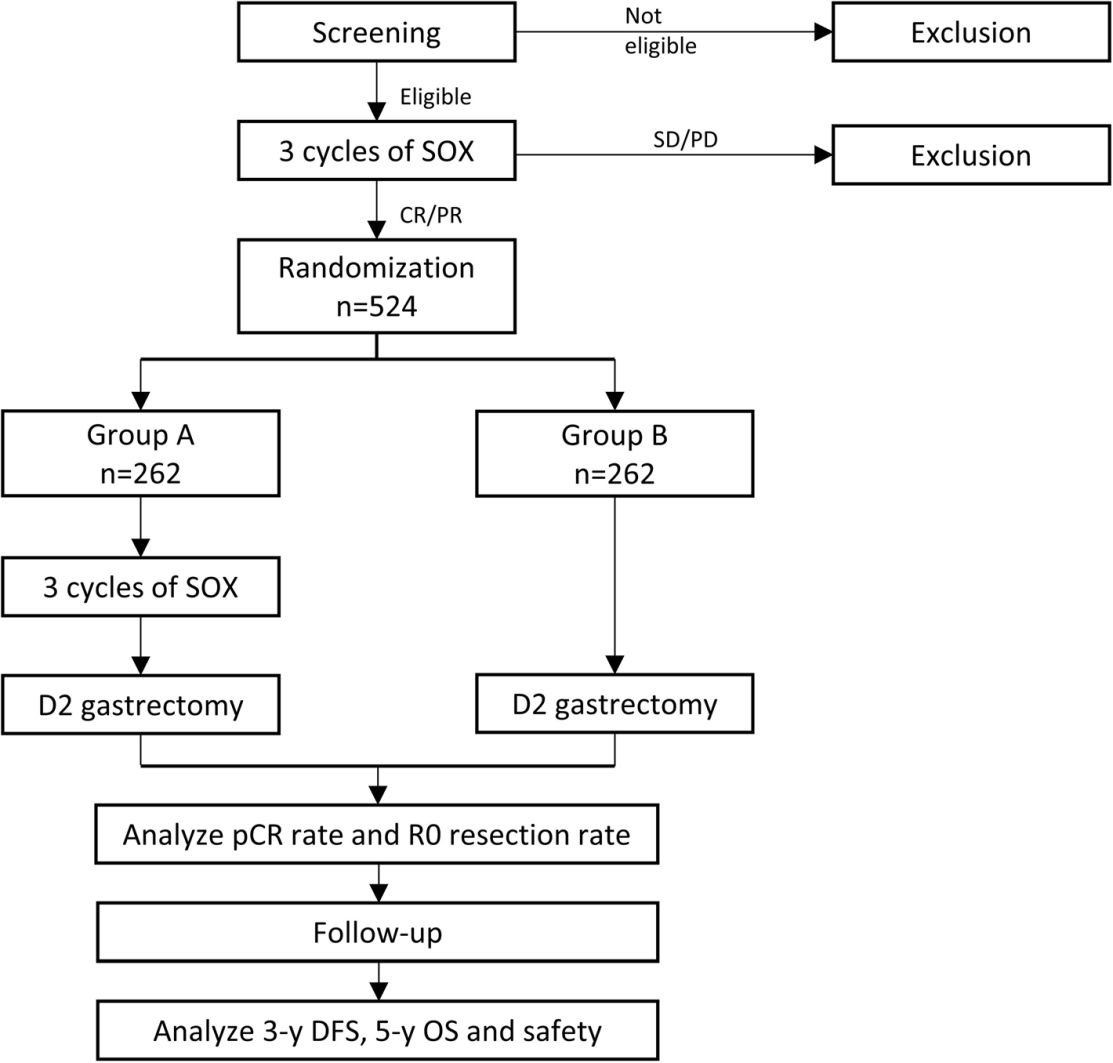
Study Flow Chart

Pre-enrolled patients will receive three cycles of SOX. Then, tumor response evaluation will be carried out according to the Response Evaluation Criteria for Solid Tumors (RECIST) 1.1 [19]. Those who demonstrate stable disease (SD) or progressive disease (PD) will be excluded. Patients achieving complete response (CR) or partial response (PR) will be enrolled and assigned into either group A (six cycles of neoadjuvant chemotherapy with SOX) for another three cycles of SOX followed by D2 surgery or group B (three cycles of neoadjuvant chemotherapy with SOX) for D2 surgery. Figure 1 showed the study flow chart. The trial has been approved by the ethics committee of the Chinese PLA General Hospital, Beijing, China and registered prospectively in the World Health Organization International Clinical Trials Registry Platform (WHO ICTRP) with registration number ChiCTR1900023293 on May 21st, 2019. All patients are required to sign the written informed consent.

Primary endpoint: Pathological complete response rate (pCR%) based on Ryan’s 0–3 pathological tumor regression grade (TRG) system [20].

Secondary endpoints: R0 resection rate, three-year disease-free survival (3-y DFS), five-year overall survival (5-y OS) and safety.

The study started in October 2019. Local ethics committee approvals are expected in October 2020, the enrollment of the first patient in November 2020, the last patient completing the research in November 2027, database lock in June 2027 and publication in June 2028.

### Patient eligibility

#### Inclusion criteria

- Non-bedridden, aged 18 to 70 years old;
- Eastern Cooperative Oncology Group (ECOG) score is 0 to 1;
- Histologically confirmed gastric adenocarcinoma;
- Have evaluable lesions based on RECIST 1.1;
- Stage III (cT3-4aN1-3 M0, American Joint Committee on Cancer (AJCC) TNM staging system 8th edition) gastric cancer confirmed by enhanced computer tomography (enhanced CT) and laparoscopic exploration (endoscopic ultrasonography (EUS) and magnetic resonance imaging (MRI) if necessary);
- The research center and the surgeon have the ability to complete standard D2 radical gastrectomy, and the gastrectomy can be tolerated by the patient;
- Laboratory test criteria: peripheral blood hemoglobin (Hb) ≥ 90 g/L, neutrophil absolute count ≥ 3× 10^9^ /L, platelet count (PLT) ≥ 100× 10^9^ /L, alanine aminotransferase (ALT) and aspartate aminotransferase (AST) ≤ 2.5 times the upper limit of normal (ULN), total bilirubin ≤ 1.5×ULN, serum creatinine (SCr) ≤ 1.5×ULN, and serum albumin (ALB) ≥ 30 g/L;
- Patients with heart disease, echocardiogram showing that the left ventricular ejection fraction ≥ 50%, electrocardiogram (ECG) is basically normal within 4 weeks before operation and with no obvious symptoms are acceptable;
- There is no serious underlying disease that could lead to an expected life expectancy < 5 years;
- Willing to sign the informed consent for participation and publication of results.

#### Exclusion criteria

- Pregnant or lactating women;
- Positive pregnancy test for women in childbearing age. Menopausal women without menstruation for at least 12 months can be regarded as women with no possibility of getting pregnant;
- Refusal of birth control during the study;
- Prior chemotherapy, radiotherapy or immunotherapy;
- History of other malignant diseases in the last 5 years (except for cervical carcinoma in situ);
- History of uncontrolled central nervous system diseases, which could influence the compliance;
- History of severe liver diseases (Child-Pugh class C), renal diseases (endogenous creatinine clearance rate (Ccr) ≤ 50 ml/min or SCr > 1.5 ULN) or respiratory diseases; Uncontrolled diabetes and hypertension; Clinically severe heart disease, such as symptomatic coronary heart disease, New York Heart Association (NYHA) class II or more severe congestive heart failure, uncontrolled arrhythmia requiring drug intervention, or a history of myocardial infarction in the last 6 months;
- History of dysphagia, complete or partial gastrointestinal obstruction, active gastrointestinal bleeding and gastrointestinal perforation;
- On steroid treatment after organ transplant;
- With uncontrolled severe infections;
- Known dihydropyrimidine dehydrogenase deficiency (DPD);
- Anaphylaxis to any research drug ingredient;
- Known peripheral neuropathy (> NCI-CTC AE 1). Patients with only disappearance of deep tendon reflex need not to be excluded.

### Grouping

All eligible patients will be registered, pre-enrolled and receive three cycles of SOX. After tumor response evaluation using RECIST 1.1, patients achieving CR or PR will be enrolled [19]. Then randomization without stratification will be carried out by computer generated allocation using IBM SPSS Statistics 22, and enrolled patients will be randomly assigned (1:1) to Arm A or Arm B.

Arm A: Patients will be pre-enrolled and receive three cycles of SOX. After randomization, patients in Arm A will receive three more cycles of SOX (six cycles of neoadjuvant chemotherapy with SOX in total) followed by D2 gastrectomy. After which postoperative chemotherapy will not be recommended.

Arm B: Patients will be pre-enrolled and receive three cycles of SOX. After randomization, patients in Arm B will receive D2 gastrectomy (three cycles of neoadjuvant chemotherapy with SOX in total). After which postoperative chemotherapy using SOX will be recommended.

### Intervention

#### Laparoscopic exploration

Laparoscopic exploration is to detect occult peritoneal metastases and inspect the primary lesion, liver, diaphragm, pelvic organs, bowel and omentum according to the standard requirements reported before [21]. Patients with any patterns of distant metastases, suggestive of distant metastasis (M1), will be excluded from the trial and recommended to a multi-disciplinary team (MDT) for further treatment.

#### Surgery

A standard D2 radical open or laparoscopic gastrectomy will be planned 3–4 weeks after the last cycle of chemotherapy. Laparoscopic gastrectomy will be recommended. The extent of gastric resection and lymphadenectomy will be performed as per the treatment guidelines [22]. Reconstruction after gastrectomy will be decided by the surgeon. All operations will be performed by well trained and experienced surgical team to guarantee the quality of surgery, including harvesting more than 16 lymph nodes. The surgeon will determine if D2 lymphadenectomy is completed and photos of the surgical field after gastrectomy will be monitored and reviewed centrally.

#### Neoadjuvant chemotherapy

The preoperative SOX chemotherapy consists of three-week cycles of intravenously administered oxaliplatin 130 mg/m^2^ on day 1 and orally administered S-1 40–60 mg twice a day (BID) on day 1 to 14. The dose of S-1 depends on body surface area (BSA): 40 mg BID for BSA < 1.25 m^2^; 50 mg BID for 1.25 m^2^ < BSA < 1.5 m^2^; 60 mg BID for BSA > 1.5 m^2^. Day 15 to day 21 is the rest period.

### Tumor response and toxicity criteria

Lesions will be evaluated according to the RECIST 1.1 criteria after the third and the sixth cycle of SOX by enhanced CT, EUS, MRI as needed [19]. Toxicities are measured according to National Cancer Institute Common Toxicity Criteria for Adverse Event (NCI-CTC AE), version 4.0. AE will be recorded in the AE report form. Serious adverse events (SAE) are defined according to the rules of good clinical practice (GCP) and will be reported to the lead center within one working day, after which other centers will be notified promptly.

### Follow-up

After treatment, patients will be examined every 6 months for 5 years, then every 12 months for life. The follow-up will include enhanced CT/MRI, abdominal ultrasound, chest X-ray, physical and laboratory examination, according to the schedule (Table 1).

**Table 1:** Follow-up schedule

Postoperative (years)	1			2		3		4		5	
Postoperative (months)	1	6	12	18	24	30	36	42	48	54	60
Body weight Complete blood count Biochemical test Tumor markers	√	√	√	√	√	√	√	√	√	√	√
Abdominal ultrasound		√	√	√	√	√	√	√	√	√	√
Abdominal CT/MRI	√	√	√	√	√	√	√	√	√	√	√
Chest X-ray			√		√		√		√		√

### Sample size calculation

The primary endpoint of this trial is pCR rate. Based on the data from previous study, after three cycles of SOX, the PCR rate is about 7% (*p*_*0*_), and we estimated that with six cycles of SOX, the PCR rate can be increased to 15% (*p*). Based on the Z test, the null and alternative hypotheses are *H*_*0*_: *p*=*p*_*0*_; *H*_*1*_: *p*≠*p*_*0*_. With the statistical power of 0.8 (β=0.2), the type I error rate of 0.05 (α=0.05), when *Z* > marginal value, the *H*_*0*_ will be rejected and *H*_*1*_ will be accepted. Using the following formulas with the sampling ratio is 1:1 and the drop-out rate of 10%, the sample size is set at 262 per arm [23].

$$ n=p\left(1-p\right){\left(\frac{z_{1-\frac{\alpha }{2}}+{z}_{1-\beta }}{p-{p}_0}\right)}^2 $$

Each participant center takes 400 to 1000 cases of gastric cancer per year and about 2% of patients can be enrolled. Thus recruitment can be completed in 2 years.

### Statistical analysis

The pCR rate is defined as the rate of patients achieving pCR. The R0 resection rate is defined as the rate of R0 resection. The DFS is defined as the period from the time of surgery to recurrence or death. The OS is defined as the period from the time of surgery to death or last follow-up. Chi-square test will be used to compare patients’ characteristics, pCR rate and R0 resection rate between two arms. Survival curves will be estimated by the Kaplan-Meier method. Survival rate of DFS and OS will be compared respectively between two arms using a two-sided log-rank test. Both intention-to-treatment analysis (ITT, patients who receive any treatment after enrollment) and per-protocol analysis (PP, patients who receive all preoperative chemotherapy and surgery) will be performed. Missing data will be processed using multiple imputation. For loss of follow-up, ITT analysis and sensitivity analysis will be performed respectively.

### Management and quality control

Each participating surgical team performs over 200 gastrectomies with D2 lymphadenectomies. Each researcher has over 20 years of professional experience. The role of clinical research organization (CRO) is filled by Beijing Sinocro PharmaScience Co.,Ltd. The lead center and CRO will organize periodical training in standard operating procedure (SOP) of gastroscopy, enhanced CT, EUS, MRI, pathological examination, staging and response evaluation. Local PI will be responsible for staging, observation of D2 completion and identification of eligible patients. To address interrater disparities, three researchers will perform the evaluation. The central review for observation and evaluation will be conduct.

CRO will appoint and authorize clinical research associates (CRA) as monitors throughout the trial according to the SOP. CRA are responsible for regularly monitoring case report forms (CRF), ensuring compliance with the protocol and GCP regulations, and checking completeness, consistency and accuracy of data. Researchers will work with CRA to ensure that any problems can be solved. Through the Electronic Data Capture System (EDC), the lead center will have the right to obtain all data and report the results. Other participating centers will only have the right to obtain the data from their own centers.

The test drugs will be purchased by the patients at their own expense according to medical orders, and the pharmacies of participating centers will be responsible for distributing them. The patients will take the medicine and make records in the patient diaries, which will be regularly monitored by CRA.

National Medical Products Administration of China and Human Genetic Resource Administration of China will inspect the trial and fulfillment of legal requirements.

## Discussion

The RESONANCE-II study was conducted to evaluate the efficacy and safety of different cycles of SOX as neoadjuvant chemotherapy for patients with locally advanced gastric cancer. The primary endpoint of the study is the rate of pathological complete response. A study evaluated the histopathological tumor regression in 480 gastric cancer patients who received surgery after neoadjuvant chemotherapy. The results showed that complete or subtotal tumor regression was an independent prognostic factor for survival, which suggested that pathological complete regression could be regarded as a proper endpoint for neoadjuvant trials in gastric cancer [24]. Moreover, our study aims to evaluate the efficacy of preoperative chemotherapy. Using pCR rate as the primary endpoint instead of survival could reduce the influence of surgery and postoperative chemotherapy on the endpoint. Also, less time will be required to get the primary results. Therefore, we chose pCR rate as the primary endpoint. However, to illustrate the relation between tumor regression and survival thoroughly, further research is still needed.

In our study, eligible patients will be pre-enrolled and receive 3 cycles of SOX. Those who achieve CR or PR will be enrolled and considered as subjects with cancer that is sensitive to SOX chemotherapy. As for patients achieve SD or PD, they will be considered as subjects with tumors that are not sensitive to SOX chemotherapy and will be excluded. The purpose of this setting is to answer the question of whether more cycles of preoperative chemotherapy should be applied when it is effective in short-term application. When preoperative chemotherapy shows little effectiveness, other treatments will be recommended instead of continuing to apply chemotherapy. In addition, pre-enrollment can ensure the completion of chemotherapy after randomization and reduce the early termination of chemotherapy due to poor efficacy. Therefore, in our opinion, the design is appropriate.

The total number of cycles of preoperative and postoperative chemotherapy in most trials is about six to eight [8–10]. In this study, patients in group A who receive 6 cycles of neoadjuvant chemotherapy can be considered to have completed all perioperative chemotherapy. It will be recommended that these patients could not receive adjuvant chemotherapy with close monitoring after surgery if there is no evidence of disease progression. Relevant results of group A may suggest the feasibility of applying only preoperative chemotherapy in gastric cancer.

To avoid bias, uniform inclusion and exclusion criteria will be applied and randomization will be performed. To prevent loss to follow-up, investigators will keep timely and effective contact with patients and provide a user-friendly mobile phone application for patients to contact investigators and record medications. All radiologists, endoscopists and pathologists will not participate in any process relevant to grouping or intervention and they will not have access to any chemotherapy-related or surgery-related records. SOPs will be defined and used with sufficient training. The CRO will monitor throughout the project.

To the best of our knowledge, this study is the first phase III randomized trial to compare the cycles of neoadjuvant chemotherapy using SOX for resectable locally advanced cancer. Due to the relatively high efficacy and safety of SOX neoadjuvant chemotherapy, it can be widely used for treatment of resectable locally advanced gastric cancer. We hope that the results of this trial can provide theoretical basis for setting an optimal duration of neoadjuvant chemotherapy.

## Data Availability

The data and materials of the study will be made available on request.

## Abbreviations

SOX: S-1 combined with oxaliplatin
WHO ICTRP: World health Organization international clinical trails registry platform
XELOX: Capecitabine and oxaliplatin
ECF: Epirubicin, cisplatin and fluorouracil
CF: Fluorouracil plus cisplatin
FLOT: Fluorouracil plus leucovorin, oxaliplatin and docetaxel
ECF/ECX: Epirubicin, cisplatin and fluorouracil or capecitabine
SC: S-1 plus cisplatin
PC: paclitaxel plus cisplatin
RECIST: Response evaluation criteria for solid tumors
SD: Stable disease
PD: Progressive disease
CR: Complete response
PR: Partial response
pCR%: The rate of pathological complete response
3-y DFS: Three-year disease free survival
5-y OS: Five-year overall survival
ECOG: Eastern cooperative oncology group
AJCC: American joint committee on cancer
CT: Computer tomography
EUS: Endoscopic ultrasonography
MRI: Magnetic resonance imaging
Hb: Hemoglobin
PLT: Platelet count
ALT: Alanine aminotransferase
AST: Aspartate aminotransferase
SCr: Serum creatinine
ALB: Serum albumin
ULN: Upper limit of normal
Ccr: Creatinine clearance rate
ECG: Electrocardiogram
DPD: Dihydropyrimidine dehydrogenase deficiency
BID: Bis in die (twice a day)
BSA: Body surface area
NYHA: New York heart association
NCI-CTC AE: National cancer institute common toxicity criteria for adverse event
GCP: Good clinical practice
ITT: Intention-to-treat
PP: Per-protocol
NCCN: National comprehensive cancer network
CSCO: Chinese society of clinical oncology
TRG: Tumor regression grade
CRO: Clinical research organization
SOP: Standard operating procedure
CRA: Clinical research associate
CRF: Case report form
EDC: Electronic data capture

## References

[1] Bray F, Ferlay J, Soerjomataram I (2018). Global cancer statistics 2018: GLOBOCAN estimates of incidence and mortality worldwide for 36 cancers in 185 countries. CA Cancer J Clin.

[2] Chen W, Sun K, Zheng R (2018). Cancer incidence and mortality in China, 2014. Chin J Cancer Res.

[3] Chun N, Ford JM (2012). Genetic testing by cancer site: stomach. Cancer J.

[4] Nakagawa N, Kanda M, Ito S (2018). Pathological tumor infiltrative pattern and sites of initial recurrence in stage II/III gastric cancer: propensity score matching analysis of a multi-institutional dataset. Cancer Med.

[5] Kim JH, Lee HH, Seo HS (2018). Borrmann type 1 Cancer is associated with a high recurrence rate in locally advanced gastric Cancer. Ann Surg Oncol.

[6] Sasako M, Sakuramoto S, Katai H (2011). Five-year outcomes of a randomized phase III trial comparing adjuvant chemotherapy with S-1 versus surgery alone in stage II or III gastric cancer. J Clin Oncol.

[7] Bang YJ, Kim YW, Yang HK (2012). Adjuvant capecitabine and oxaliplatin for gastric cancer after D2 gastrectomy (CLASSIC): a phase 3 open-label, randomised controlled trial. Lancet..

[8] Cunningham D, Allum WH, Stenning SP (2006). Perioperative chemotherapy versus surgery alone for resectable gastroesophageal cancer. N Engl J Med.

[9] Ychou M, Boige V, Pignon JP (2011). Perioperative chemotherapy compared with surgery alone for resectable gastroesophageal adenocarcinoma: an FNCLCC and FFCD multicenter phase III trial. J Clin Oncol.

[10] Al-Batran SE, Homann N, Pauligk C (2019). Perioperative chemotherapy with fluorouracil plus leucovorin, oxaliplatin, and docetaxel versus fluorouracil or capecitabine plus cisplatin and epirubicin for locally advanced, resectable gastric or gastro-oesophageal junction adenocarcinoma (FLOT4): a randomised, phase 2/3 trial. Lancet.

[11] Schuhmacher C, Gretschel S, Lordick F (2010). Neoadjuvant chemotherapy compared with surgery alone for locally advanced cancer of the stomach and cardia: European Organisation for Research and Treatment of Cancer randomized trial 40954. J Clin Oncol.

[12] Oh SY, Kwon HC, Jeong SH (2012). A phase II study of S-1 and oxaliplatin (SOx) combination chemotherapy as a first-line therapy for patients with advanced gastric cancer. Investig New Drugs.

[13] Koizumi W, Takiuchi H, Yamada Y (2010). Phase II study of oxaliplatin plus S-1 as first-line treatment for advanced gastric cancer (G-SOX study). Ann Oncol.

[14] Xiao C, Qian J, Zheng Y (2019). A phase II study of biweekly oxaliplatin plus S-1 combination chemotherapy as a first-line treatment for patients with metastatic or advanced gastric cancer in China. Medicine (Baltimore).

[15] Li T, Chen L (2011). Efficacy and safety of SOX regimen as neoadjuvant chemotherapy for advanced gastric cancer. Zhonghua Wei Chang Wai Ke Za Zhi.

[16] NCCN Clinical Practice Guidelines in Oncology Gastric Cancer Version 1. 2020. https://www.nccn.org/professionals/physician_gls/pdf/gastric.pdf. Accessed 10^th^ June 2020.

[17] Wang FH, Shen L, Li J (2019). The Chinese Society of Clinical Oncology (CSCO): clinical guidelines for the diagnosis and treatment of gastric cancer. Cancer Commun (Lond).

[18] Yoshikawa T, Morita S, Tanabe K (2016). Survival results of a randomised two-by-two factorial phase II trial comparing neoadjuvant chemotherapy with two and four courses of S-1 plus cisplatin (SC) and paclitaxel plus cisplatin (PC) followed by D2 gastrectomy for resectable advanced gastric cancer. Eur J Cancer.

[19] Eisenhauer EA, Therasse P, Bogaerts J (2009). New response evaluation criteria in solid tumours: revised RECIST guideline (version 1.1). Eur J Cancer.

[20] Ryan R, Gibbons D, Hyland JM (2005). Pathological response following long-course neoadjuvant chemoradiotherapy for locally advanced rectal cancer. Histopathology.

[21] Li H, Zhang Q, Chen L (2017). Role of diagnostic laparoscopy in the treatment plan of gastric cancer. Zhonghua Wei Chang Wai Ke Za Zhi..

[22] Japanese Gastric Cancer Association. Japanese gastric cancer treatment guidelines 2018 (5^th^ edition). Gastric Cancer. 2020. 10.1007/s10120-020-01042-y.

[23] Chow S, Shao J, Wang H (2008). Sample size calculations in clinical research.

[24] Becker K, Langer R, Reim D (2011). Significance of histopathological tumor regression after neoadjuvant chemotherapy in gastric adenocarcinomas: a summary of 480 cases. Ann Surg.

